# Comparative Transcriptomics Indicates a Role for *SHORT VEGETATIVE PHASE* (*SVP*) Genes in *Mimulus guttatus* Vernalization Response

**DOI:** 10.1534/g3.115.026468

**Published:** 2016-02-25

**Authors:** Jill C. Preston, Jinshun Zhong, Meghan McKeown, Meghan den Bakker, Jannice Friedman

**Affiliations:** * Department of Plant Science, University of Vermont, Burlington, Vermont 05405; † Department of Biology, Syracuse University, New York 13244

**Keywords:** flowering time, local adaptation, *Mimulus guttatus*, photoperiod, *SHORT VEGETATIVE PHASE*, vernalization

## Abstract

The timing of reproduction in response to variable environmental conditions is critical to plant fitness, and is a major driver of taxon differentiation. In the yellow monkey flower, *Mimulus guttatus*, geographically distinct North American populations vary in their photoperiod and chilling (vernalization) requirements for flowering, suggesting strong local adaptation to their surroundings. Previous analyses revealed quantitative trait loci (QTL) underlying short-day mediated vernalization responsiveness using two annual *M. guttatus* populations that differed in their vernalization response. To narrow down candidate genes responsible for this variation, and to reveal potential downstream genes, we conducted comparative transcriptomics and quantitative PCR (qPCR) in shoot apices of parental vernalization responsive IM62, and unresponsive LMC24 inbred lines grown under different photoperiods and temperatures. Our study identified several metabolic, hormone signaling, photosynthetic, stress response, and flowering time genes that are differentially expressed between treatments, suggesting a role for their protein products in short-day-mediated vernalization responsiveness. Only a small subset of these genes intersected with candidate genes from the previous QTL study, and, of the main candidates tested with qPCR under nonpermissive conditions, only *SHORT VEGETATIVE PHASE* (*SVP*) gene expression met predictions for a population-specific short-day-repressor of flowering that is repressed by cold.

The transition to flowering is a crucial decision that requires synchronization with favorable environmental conditions. Plants have evolved the ability to perceive and respond to both internal and external signals to maximize fitness by flowering at appropriate times. In the northern and southern temperate zones, flowering time is often determined by seasonal fluctuations in photoperiod, and variation in the duration of winter chilling (vernalization) (Hut *et al.* 2013; Preston and Sandve 2013). Furthermore, photoperiod and vernalization responsiveness often vary within a species, and strong latitudinal clines suggest that local adaptation in flowering time genes is common (Samis *et al.* 2012; Van Dijk and Hautekèete 2014; Woods *et al.* 2014).

The genetic basis of photoperiod- and vernalization-induced flowering is best understood in temperate annuals, such as *Arabidopsis thaliana* (Brassicaceae) and *Triticum aestivum* (wheat, Poaceae). In *A. thaliana*, long days promote the expression of the circadian oscillator gene *GIGANTEA* (*GI*) in leaves, resulting in an increase in the transcription of *CONSTANS* (*CO*) (Mizoguchi *et al.* 2005; Sawa *et al.* 2007). CO is a positive regulator of the flowering pathway integrator gene *FLOWERING LOCUS T* (*FT*), whose protein product provides the florigen signal from leaves to the shoot apical meristem, resulting in rapid flowering (Suárez-López *et al.* 2001; Song *et al.* 2013). Whereas the long-day photoperiod pathway is strongly conserved across distantly related species, the vernalization pathway has evolved convergently, in many cases through the cooption of distinct genes (Amasino and Michaels 2010; Preston and Sandve 2013).

In *A. thaliana*, precocious flowering under both long and short days is suppressed by the action of *SHORT VEGETATIVE PHASE* (*SVP*), *FLOWERING LOCUS C* (*FLC*), and the microRNA *miR156*. SVP is a MADS-box protein that represses flowering by binding to the CArG boxes of *FT* in the phloem, and *SUPPRESSOR OF OVEREXPRESSION OF CONSTANS 1* (*SOC1*) in the shoot apical meristem (Li *et al.* 2008; Gregis *et al.* 2013). *FLC* is the major *A. thaliana* vernalization gene that blocks transcription of *FT* in the phloem and shoot apices (Searle *et al.* 2006), and is repressed by cold responsive genes such as *VERNALIZATION 1* (*VRN1*) (Levy *et al.* 2002; Mylne *et al.* 2006). In contrast to *SVP* and *FLC*, which are unaffected or upregulated with age, *miR156* transcripts are abundant only in young plants. Prior to attaining flowering competency, *miR156* represses the expression of *SQUAMOSA PROMOTER BINDING PROTEIN LIKE* (*SPL*) genes, resulting in maintenance of the juvenile phase (Yang *et al.* 2011). In perennial *Arabis alpina* (Brassicaceae) age-dependent changes in *miR156* partially account for its late-stage vernalization response (Bergonzi *et al.* 2013). However, whether modifications in the regulation of the aforementioned flowering time genes can account for eudicot flowering time variation outside Brassicaceae is generally not well understood (but see Pin *et al.* 2010; Vogt *et al.* 2014).

The yellow monkey flower (*Mimulus guttatus*, Phrymaceae) is a model system for understanding the genetic basis of local adaptation, because the species shows incredible variation in life history, development, and physiological traits, and occupies a broad range of habitats and edaphic conditions (Twyford *et al.* 2015). Previous studies have shown that populations of *M. guttatus* vary considerably in their critical photoperiod- and vernalization-induced flowering response (Friedman and Willis 2013). For example, in an annual population from the Californian Coastal Range (LMC), flowering typically occurs with 10–11 hr of daylight, whereas a high-altitude annual population from the Oregon Cascades (IM) requires at least 14 hr of daylight to flower (Friedman and Willis 2013). Remarkably, and unlike other vernalization responsive species described, after several weeks of 8-hr days (short-days), the IM Oregon alpine plants are unable to flower, even after transfer back to 16-hr-long-days unless they receive a vernalization treatment (Friedman and Willis 2013). In contrast, California LMC plants flower readily upon transfer back to long-day conditions, and thus do not require vernalization to flower (Friedman and Willis 2013). The adaptive significance of variation in the photoperiod-dependent vernalization response is still unknown, although it may be related to the timing of germination, and transition to flowering in different environments.

Quantitative trait locus (QTL) mapping of F_2_ recombinants between the aforementioned IM and LMC populations previously revealed two major loci on linkage groups (LG) 8 and 11, that underlie differences in the photoperiod-mediated vernalization response (Friedman and Willis 2013). Together, these QTL regions harbor 435 (LG 8) and 592 (LG 11) predicted genes, 20 of which are annotated in Phytozome v9.1 as flowering time genes based on homology with genes from *A. thaliana* (Goodstein *et al.* 2012) (Supplemental Material, Table S1). Here, we use comparative transcriptome analyses to identify shared and differential responses between populations grown under different environmental conditions. Based on the assumption that differential gene expression indicates a functional involvement of the encoded proteins in mediating flowering responses, we identified candidate genes that intersect with previous QTL mapping. We then contrast gene expression of these candidate genes at various time points, and between populations in permissive *vs.* nonpermissive conditions, thus revealing a handful of candidate genes underlying the evolution of short-day-mediated vernalization responsiveness.

## Materials and Methods

### Plant materials

Seed material for this experiment originated from an annual vernalization responsive *M. guttatus* population located in Oregon’s Western Cascades (IM: 44°24’059”, –122°08’946”, 1430 m a.s.l.), and from an annual vernalization unresponsive population located about 50 km from the coast in California (LMC: 38**°**51’50”, –123**°**05’02”, 306m a.s.l.). We used seed from a single highly inbred line from each population (IM62 and LMC24; these were the same lines previously used for QTL mapping). We sowed seeds in 2” pots filled with moist Fafard 4P potting mix, and stratified them in the dark at 4° for 1 wk at Syracuse University.

### Experimental design

**Figure 1:**
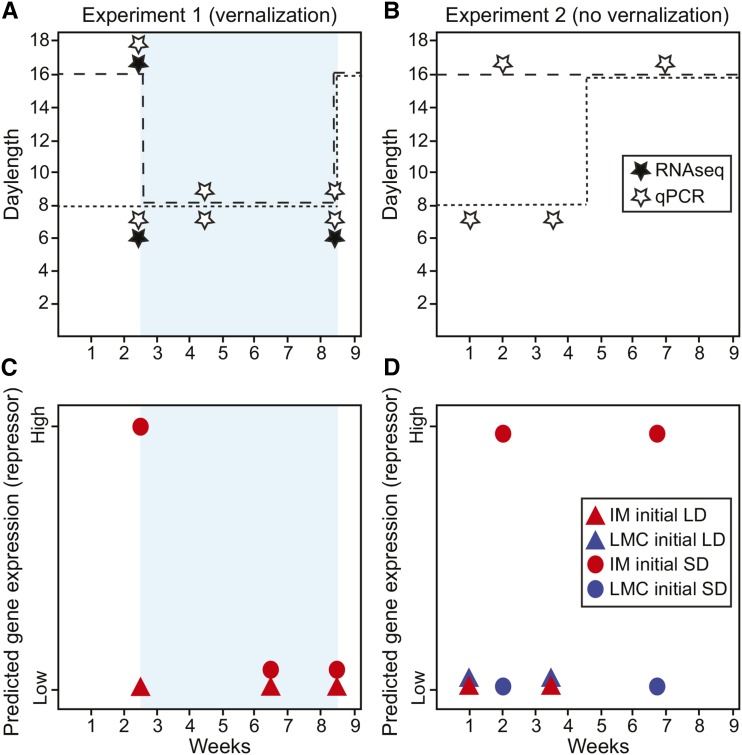
Diagram of experimental design and sampling strategy for the photoperiod experiments with (experiment 1) and without (experiment 2) vernalization. (A) IM62 and LMC24 inbred plants were grown for 2.5 wk under 21° 16 h long days (dashed lines) or repressive 8 h short days (dotted lines), and then moved to short days at 4° (light blue shading) for 6 wk followed by long days at 21° to induce flowering. Shoot apical meristems were harvested for RNA just prior to vernalization (short day or long day 17 d “precold”), during vernalization, and at the end of vernalization (6 wk long day “postcold” with initial short days or long days) for RNAseq and/or qPCR analyses, as indicated by closed or open symbols, respectively. (B) IM62 and LMC24 inbred plants were grown for 4.5 wk under 21° 16 h long days (dashed lines) or repressive 8 h short days (dotted lines), and then moved to 21° long days until flowering or termination of the experiment. In contrast to experiment 1, shoot apical meristems were harvested for qPCR (open symbols) at developmentally similar time points based on exposure to 120 and 384 light hrs. This corresponded to 1 wk and 3.5 wk for long-day plants, and 2 wk and 7 wk for plants initially given short days. (C) Predictions for gene expression in experiment 1 for candidate IM62 genes (red symbols) that repress flowering; no specific predictions were made for LMC24. (D) Predictions for gene expression for experiment 2 for candidate IM62 (red symbols) and LMC24 (blue symbols) genes that repress flowering. Opposite predictions to those shown in (C) and (D) can be made for genes that promote flowering (not shown). In panels (C) and (D) the different shapes refer to whether plants received initial short days (SD) or long days (LD) in their respective experiments.

We conducted two independent experiments (with and without vernalization), each consisting of two treatments (initial long-day or short-day) (Figure 1). For experiment 1 (Figure 1A), we randomly assigned 200 newly germinated seedlings of each inbred line to one of two plant growth chambers for 2.5 wk: long-day warm (16 hr daylength, constant 21° temperature), or short-day warm (8 hr daylength, constant 21° temperature). After 2.5 wk, we moved all plants to a common short-day cold (8 hr daylength, constant 4° temperature) chamber for 6 wk of vernalization. Following vernalization, we reassigned plants to a long-day warm (16 hr daylength, constant 21° temperature) chamber until flowering. For experiment 2 (Figure 1B), we randomly assigned 200 newly germinated seedlings of each inbred line to one of two plant growth chambers: long-day warm or short-day warm, as previously described, for 4.5 wk. We then transferred plants to a common long-day warm chamber until flowering, death, or termination of the experiment. For both experiments, we rotated plants and chamber conditions, and randomized plants within treatment every 3 d to reduce unintentional effects associated with each chamber or position. We counted leaf number and length of the oldest leaf at every tissue harvest time (see next section), and recorded the number of days to flowering postgermination. We analyzed leaf number, leaf length, and days to flowering separately using general linear models in R 3.0.2 (R Core Team 2013), with photoperiod, population, and time (leaf number measures only), treated as categorical independent variables. We compared pairwise differences in response variables using a Tukey test in R 3.0.2 (R Core Team 2013).

To determine the duration of short-days necessary to inhibit flowering in the vernalization responsive population, we germinated 100 additional IM62 seedlings under short-day warm conditions, transferring three plants to long-day warm conditions every 3 d up to 27 d, and measured days to flowering (Figure S1). Similarly, to assess the period of vernalization required to break short-day-inhibition of flowering, we transferred 28-d short-day warm grown IM62 seedlings to short-day cold conditions, and moved three plants every 3 d, up to 42 d, to long-day warm conditions, and measured days to flowering (Figure S1).

### Tissue collection, RNA extraction, and cDNA synthesis

We destructively harvested shoot apical meristems with surrounding leaf tissue from 10 individuals per time point/treatment/line (referred to as a cell henceforth), and shipped samples on dry ice to the University of Vermont for gene expression analysis. The schedule of shoot apex sampling is outlined in Figure 1. For experiment 1, we collected shoot apices from both IM62 and LMC24 plants following an initial 17 d in long-day or short-day photoperiods at 21° (“precold”), and then after 4 wk (45 d total), and 6 wk (59 d total) of short-day 4° cold (Figure 1A). For experiment 2, to correct for differences in growth between treatments, we collected shoot apices from long- and short-day chamber plants following a total of five and 16 light days (Figure 1B). Light days were calculated based on total exposure to light, with 5 d of light being equal to 15 short-days and 7.5 long-days, and 16 d of light being equal to 48 short-days and 24 long-days. We ground two replicates of two to three pooled shoot apices per cell in liquid nitrogen for RNAseq analyses (experiment 1 only), and separately ground five apices per cell for quantitative (q)-PCR analyses (experiments 1 and 2). We extracted RNA using TriReagent (Life Technologies, NY), and removed residual DNA with Turbo DNase (Life Technologies, NY) according to the manufacturers’ instructions.

We used 1 μg of each sample in RNA library preparation using TruSeq RNA Library Preparation Kit v2 (Illumina, CA) following the manufacturer’s instructions, for two times 100-bp paired-end sequencing. We assessed RNA yield and fragment insert sizes before and after library preparation, using an Agilent 2100 Bioanalyzer. We sequenced libraries on an Illumina HiSeq1000 using two Illumina flow cells, splitting all samples evenly across cells to control for lane effects. In total, we sequenced 12 libraries from experiment 1, comprising two replicates of short-day precold, long-day precold, and 6 wk cold-treated IM62 and LMC24. Library construction and RNA sequencing was carried out by the Advanced Genome Technologies Core at the University of Vermont. For qPCR analysis, we synthesized cDNA using iScript reverse transcriptase (BioRad, CA) on 1 μg template RNA and diluted 1:10 in water.

### Transcriptome assembly, annotation, and differential expression analysis

After demultiplexing, we filtered out sequencing adapters and low-quality sequences using Trimmomatic-0.33 (LEADING:20 TRAILING:20 SLIDINGWINDOW:5:20 MINLEN:40) (Bolger *et al.* 2014). We then trimmed sequencing adapters and leading low quality bases (below quality 20), and removed reads if the average quality per base in a 5-bp sliding window was below 20. Only reads from paired-end sequencing that were both longer than 40 bps, following trimming for quality, were used for later analyses. For assembly of clean reads, we used a *de novo* approach for both IM62 and LMC24 with default parameters in Trinity v2.06 (Haas *et al.* 2013). We favored this approach over assembly to the IM62 reference genome, because IM62 and LMC24 show considerable population divergence (Brandvain *et al.* 2014). This level of divergence has the potential to make direct alignment of LMC24 short reads to the reference *Mimulus* genome sequences (IM62) imprecise, likely leading to faulty counts of expression levels. Additionally, we decided against using reference-guided alignment for IM62 alone to reduce bias introduced by different transcriptome processing approaches.

Following transcriptome assembly, potential coding regions were predicted using TransDecoder v2.01 (Haas *et al.* 2013), and gene ontology (GO) categories were determined using Trinotate with follow-up reference to UniProt (The Gene Ontology Consortium 2000; Grabherr *et al.*, 2011; The UniProt Consortium 2014). To estimate the expression values of assembled transcripts, we mapped cleaned short reads from each treatment to Trinity-assembled transcripts using Bowtie 1.01 (Langmead *et al.* 2009), and RSEM (Li and Dewey 2011), with custom scripts from the Trinity package. To account for allelic variation and sequencing errors, we used predicted genes rather than isoforms for downstream analyses. Although this method may collapse closely related paralogs into single predicted genes, we assume this will not affect analyses of differential expression, since very recent paralogs are likely to be similarly expressed, subfunctionalized in expression, and/or show compensation for gene dosage effects (Rogozin 2014).

To determine which transcripts/genes were differentially expressed between treatments, we conducted pairwise comparisons in R/Bioconductor using the DeSeq2 package (Love *et al.* 2014). We binned candidate vernalization responsive genes into two categories: those that fit predictions of flowering repressors (or their downstream targets), and those that fit predictions of flowering promoters (or their downstream targets). Candidates for short day vernalization-responsive flowering repressors included IM62 genes that significantly (*adj P* < 0.05) showed three patterns (Figure 1C): 1) higher expression at 17 d precold under short-day *vs.* long-day photoperiods; 2) decreased expression from precold under short-days to postcold under long-days, with an initial period of short-days; and 3) no significant difference between 17 d precold long-day, and 6 wk postcold long-day samples given an initial period of short-days. Inclusion of the third category aimed to identify repressors that are specifically upregulated under warm short-day conditions, but downregulated by both long-days and vernalization. Candidates for vernalization-responsive flowering promoters included IM62 genes that showed the following three patterns (Figure 1D): 1) significantly lower expression at 17 precold days under short-day *vs.* long-day photoperiods; 2) increased expression from precold under short days to postcold under long days with an initial period of short-days, and 3) no significant difference between 17 d precold long-day and 6 wk postcold long-day samples given an initial period of short-days. Although interesting to compare, we had no specific predictions for expression of LMC24 genes in the vernalization experiment (experiment 1), since repressors of flowering might either be constitutively transcribed at a low level, or be downregulated in an age-dependent manner. The reverse is true for promoters of flowering.

### Candidate gene isolation and phylogenetic analysis

Of the differentially expressed candidate genes that also fell within vernalization QTL, we selected *SVP* and *FLC/MAF* annotated flowering time genes for further study. These were selected as the most promising genes based on established function as temperature-regulated repressors of flowering in *Arabidopsis* (Lee *et al.* 2013; Posé *et al.* 2013; Angel *et al.* 2015). We used assembled contig sequences to BLAST the *M. guttatus* IM62 reference genome on Phytozome (http://www.phytozome.net/), and aligned the amino acid sequences of the top matching homologs with related genes from *A. thaliana*, and other species, in Mesquite v2.75 (Maddison and Maddison 2011). To confirm predicted mRNAs from IM62, isolate orthologs from vernalization unresponsive LMC24, and detect any alternative splice variants, we designed primer pairs to amplify near full-length sequences for each gene. Amplicons were ligated and cloned into pGEM-T (Promega, WI), and four to 10 colonies were sequenced at the University of Washington High Throughput Genomics Center with T7 or SP6. We manually checked sequences of each clone for ambiguities in base calling, and aligned with homologous genomic sequences from IM62. To verify orthology/paralogy relationships between genes, we subjected each nucleotide alignment to maximum likelihood phylogenetic analysis in GARLI under a GTR + I + Γ model of evolution, with 500 bootstrap replicates (Zwickl 2006).

### qPCR validation

We used Primer 3 (Rozen and Skaletsky 2000) to design gene specific primers that exactly matched candidate *SVP*- and *FLC/MAF*-like gene sequences of the vernalization-responsive IM62 and unresponsive LMC24 populations to amplify approximately 150-bp products (Table S2). To contrast candidate K00964 *FLC/MAF*-like gene expression with other *FLC/MAF*-like genes, we also designed primers to simultaneously amplify K00960, K00958, and K00957, or K00968, G00778, K00996, and K00963.

We conducted qPCR on a StepOne real time PCR system (Life Technologies, NY) using Fast SYBR green master mix (Life Technologies, NY) according to the manufacturers’ instructions. Each primer pair was initially used to amplify a serial dilution series of cDNA from multiple experimental cells in triplicate to calculate amplification efficiencies (Table S2). We discarded primers and designed new ones if efficiency values were below 90%, negative controls showed primer dimer peaks, samples generated multiple peaks, or sequencing failed to confirm specificity. After correcting for primer efficiency, we normalized cycle threshold (*C*_t_) values in target tissues using the geomean of two housekeeping genes, *EF1*α and *UBQ5*, as previously described (Scoville *et al.* 2011). We calculated the mean and standard deviation for four to five biological replicates, each with three technical replicates. We compared log-transformed relative gene expression for each candidate gene for experiments 1 and 2 using the ANOVA function in R 3.0.2 (R Core Team 2013), with photoperiod, population, and time, treated as categorical independent variables. We removed interaction terms by stepwise backward elimination when they were not significant. We assessed relevant *a priori* pairwise contrasts of gene expression according to a one-tailed Tukey test.

### Data availability

Seed is available on request. Table S1 contains Phytozome IDs of flowering time gene candidates identified in Friedman and Willis (2013). Table S2 provides primers used for qRT-PCR. Table S3 provides a summary of general linear model results. Figure S1 shows boxplots of days to flowering with different amounts of short days and cold. Figure S2 shows heat maps for the top differentially expressed genes across all treatments. LMC24 *SVP* and *MAF* Sanger sequence data are available at GenBank, under accession numbers KP172241– KP172246. RNA-Seq data are deposited at the Sequence Read Archive in National Center for Biotechnology Information (NCBI) under BioProject number PRJNA311324.

## Results

### Effect of photoperiod on flowering time and leaf development

**Figure 2:**
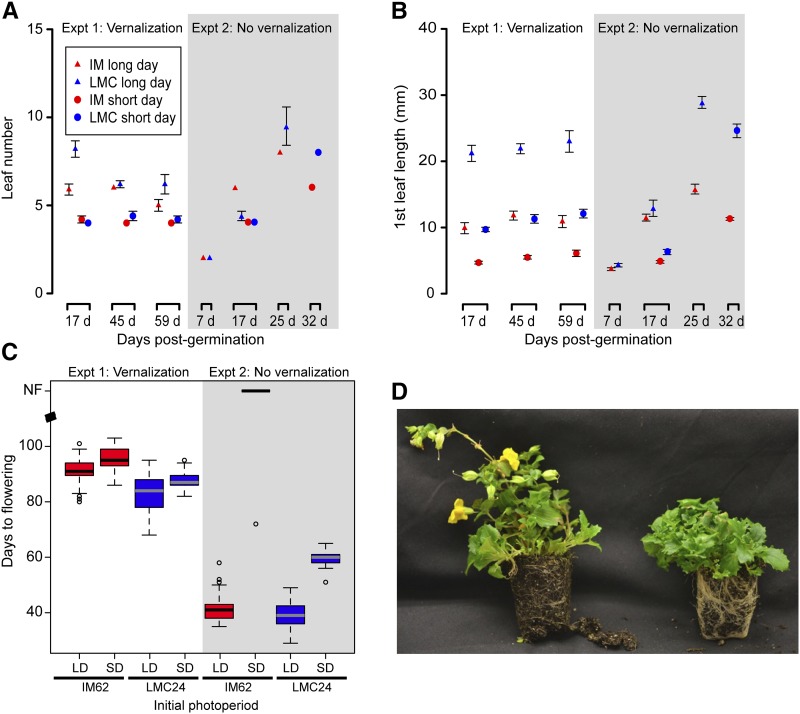
Developmental variation through time and across population and photoperiod treatments. Mean (±SE) leaf number (A) and length of the first true leaf (B) at different days postgermination in two experiments (with and without vernalization). For both experiments, the long-day photoperiod is depicted with triangles and the short-day photoperiod with circles. The vernalization-responsive IM62 is shown in red, and unresponsive LMC24 in blue. (C) Boxplot showing days to flowering for the two experiments (with and without vernalization), and the two initial photoperiod treatments (long-day or short-day). The vernalization responsive IM62 is shown in red, and unresponsive LMC24 in blue. (D) Plants from IM62 with (left) and without (right) vernalization, following initial short-day treatment.

Exposure to short days has been shown to stably repress flowering in the Oregon IM62 line, with vernalization causing derepression (Friedman and Willis 2013). To confirm this response in our experimental conditions, we assessed days to flowering, as well as leaf number, and length of the first true leaf, for plants in each treatment in the two experiments (Figure 2 and Table S3).

In experiment 1 (with vernalization), there was a significant effect of photoperiod treatment on leaf number, leaf length, and flowering (Table S3), with short-days reducing growth and delaying flowering compared to long days (Figure 2, A–C). Furthermore, there was no significant effect of time on leaf number, or leaf length, consistent with inhibition of growth during vernalization. Population had a significant effect for all three measures (Table S3), with the LMC24 plants flowering earlier, and having more rapid leaf growth and initiation than the IM62 line (Figure 2, A–C). However, there was no interaction between initial photoperiod treatment and population on days to flowering (Table S3). This lack of interaction suggests that short-days inhibit growth relative to long days in both populations; the requirement for vernalization in short-day grown IM62 plants moved to long days is thus reflective of a population-specific “memory” of short-days.

Similar to experiment 1, experiment 2 (without vernalization) showed a significant effect of photoperiod on leaf number, and photoperiod and population on first true leaf length, and days to flowering (Table S3 and Figure 2, A–C). However, unlike experiment 1, there was a significant effect of time on leaf number and leaf length. For flowering time, there was a significant interaction between photoperiod and population (Table S3). This interaction arises because, with the exception of one individual (Figure 2C), IM62 plants in the short-day photoperiod failed to flower prior to termination of the experiment at 120 d, whereas the LMC24 plants flowered after an average of 60 d. A follow-up experiment, focused on inhibition of flowering in the IM62 line, found that 6 d of the short photoperiod, and 15 d of vernalization are sufficient to inhibit and derepress flowering in 50% of plants, respectively (Figure S1).

### Read mapping and GO analysis of the top differentially expressed genes

Whole transcriptome sequencing of shoot apical meristem RNA from experiment 1 generated 183 and 195 million 100-bp Illumina reads for IM62 and LMC24 samples, respectively. Reads corresponded to 24,481 unique contigs for IM62, and 23,967 unique contigs for LMC24, with a minimum contig size of 200 bp, and an average contig length of 774 bp. For both populations, the most highly differentially expressed genes were transcripts that were either upregulated or downregulated by cold; there were fewer differences between the short day and long day treatments (Figure S2). The cold upregulated genes mostly fell into GO categories related to metabolism, defense/stress, hormone signaling, and cell wall organization, whereas the cold downregulated genes mostly included those annotated as being involved in the promotion of flowering, pigment biosynthesis, defense/stress response, auxin signaling, and metabolism (Figure S2).

### Differentially expressed IM62 genes in response to short-day vernalization

**Figure 3:**
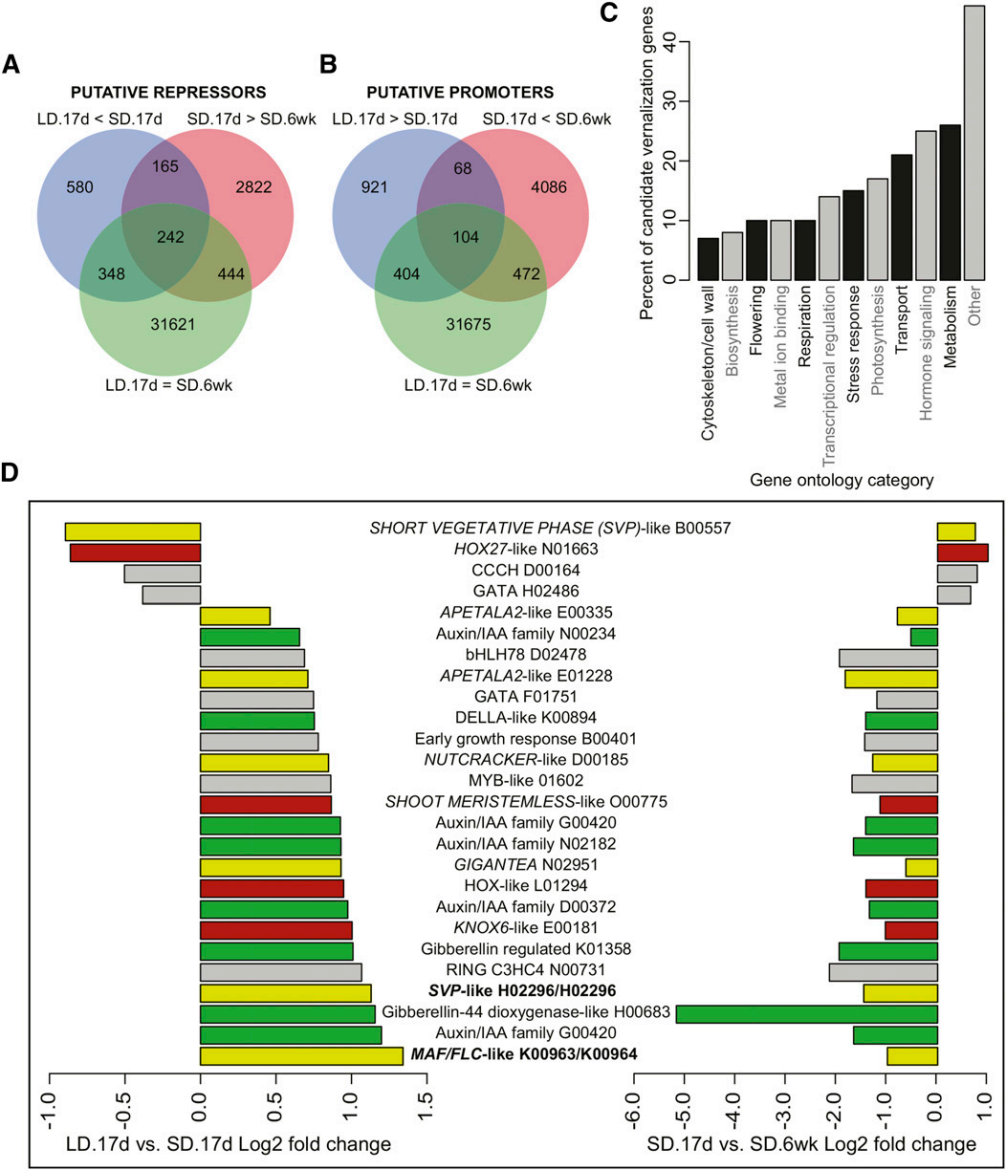
Candidate short-day mediated IM62 vernalization response genes. (A) Venn diagram showing candidate vernalization response genes that are more highly expressed (adj *P* < 0.05) in warm short-days *vs.* warm long-days (blue circle), or cold short-days (red circle), but are not differentially expressed between warm long-days and cold short-days (green circle). (B) Venn diagram showing candidate vernalization response genes that are expressed at lower levels (adj *P* < 0.05) in warm short-days *vs.* warm long-days (blue circle), or cold short-days (red circle), but are not differentially expressed between warm long-days and cold short-days (green circle). (C) Percentage of differentially expressed IM62 vernalization responsive genes in major gene ontology categories. (D) Log2 fold change of annotated transcription factors [from (A) and (B)] differentially expressed in response to photoperiod and temperature. (D) Genes found in previously described vernalization QTLs are highlighted in bold. Yellow, flowering time genes; red, meristem maintenance genes; green, hormone pathway genes; grey, other genes.

Vernalization responsiveness in IM62 is contingent on prior exposure to short days, so genes involved in this response are predicted to be downregulated (flowering repressors, and their downstream targets), or upregulated (flowering promoters, and their downstream targets) following a switch from nonpermissive (short-day warm) to permissive (long-day cold) conditions (Figure 1C). A total of 4601 predicted IM62 genes had significantly lower transcript abundance (adj *P* < 0.05) at 17 d in warm short day precold leaves, compared to the other treatments or time points. Of these, 407 were lower relative to both 17 d warm long-day precold leaves, and 6-wk long-day postcold leaves that were initially grown under short-days. Moreover, only 242 of these 407 candidate genes were expressed at similar levels between 17 d warm long-day precold leaves and 6-wk longday postcold leaves that were initially grown under short-days (Figure 3A). Similar analyses for putative promoters of flowering revealed 172 genes that were significantly more highly expressed at the 17 d precold time point in warm short-days relative to both 17 d precold warm long-days, and 6-wk long-day postcold leaves that were initially grown under short-days (adj *P* < 0.05). Of these genes, 104 were expressed at similar levels between 17 d warm long day precold leaves, and 6-wk long day postcold leaves that were initially grown under short-days (Figure 3B).

Annotation of the 414 (242 putative repressors plus 104 putative promoters) differentially expressed IM62 genes that were responsive to vernalization, revealed a bias toward metabolism, hormone signaling, transport, and photosynthesis GO categories (Figure 3C). Furthermore, 26 of the 414 genes were annotated as transcriptional regulators involved in reproductive development, hormone signaling, meristem development, or other biological functions, the former category including genes with similarity to *A. thaliana SVP*, *FLC/MAF*, *GI*, and *APETALA2* (*AP2*) (Figure 3D). To determine if any of these genes might be the causative loci underlying population differences in vernalization responsiveness, we conducted BLAST searches of the 414 IM62 candidate genes against the *M. guttatus* v2.0 genome. We found 16 and 20 genes matched sequences in the vernalization QTL on LG 8 and 11, respectively. Most of the 36 genes fell into GO categories related to metabolism, cellular transport, and membrane functioning (Table 1). However, two predicted transcripts, within the *SVP* and *MAF/FLC* clades, were annotated as being putatively involved in flowering. Further, isoform analysis, with reference to the IM62 genome (Goodstein *et al.* 2012) and our phylogenetic analyses (see below), identified the *SVP*- and *MAF/FLC*-like transcripts as comprising two recent tandem-duplicated loci: *SVP*-like H02293 and *SVP*-like H02296, and *MAF*/*FLC*-like K00963 and *MAF/FLC* K00964, respectively (Table 1).

**Table 1:** IM62 QTL candidate genes differentially expressed in response to both long days and vernalization (*adj P* < 0.05)

	Phytozome *M. guttatus* v2.0				Log2 Fold Change	
Contig	Name	Start	End	Annotation	LD17vSD17	SD17vSD6wk
LG 8						
c20784_g10	H02293/	23101975	23105300	*SHORT VEGETATIVE PHASE*	1.130	−1.467
	H02296	23123993	23126350			
c18701_g1	None	21708418	21709143	None	0.837	−2.623
c19454_g3	H02536	24794754	24797057	Alpha/beta hydrolase	0.812	−0.683
c20295_g1	H02097	20405436	20406810	Glycosyl hydrolase	0.752	−1.782
c9064_g1	H02381	23865399	23866303	Pollen protein Ole like	0.434	−1.253
c20760_g2	H02179	21575241	21578705	Ras-related GTPase	0.373	−0.729
c19039_g1	H02076	20082900	20083500	Tyrosinase	−1.447	1.692
c16419_g1	H01373	15783459	15783690	None	−0.270	0.586
c4227_g1	H00889	6689246	6689635	Voltage-dependent anion channel protein 1	−0.295	0.452
c18645_g1	H01673	17287963	17288694	20S proteasome subunit alpha 2	−0.336	0.495
c14276_g1	H00657	3759354	3759717	Ubiquitin conjugation factor E4 B	−0.370	0.659
c19572_g2	H02232	22422701	22424281	NADH dehydrogenase (ubiquinone) Fe-S protein 1	−0.370	0.757
c19477_g6	H02486	24576588	24576886	Zinc finger GATA type protein	−0.383	0.657
c18424_g1	H00553	3267601	3268039	Tryptophan synthase beta chain	−0.680	0.820
c8675_g1	H02161	21316322	21317267	GTP-binding protein TYPA/BIPA	−0.749	0.638
LG 11						
c13203_g1	K01003	14601115	14604303	None	0.918	−3.030
c15034_g1	K01358	23575699	23576697	PFAM gibberellin regulated	1.01	−1.956
c14420_g1	K00668	5002777	5003412	Acetoacetyl-CoA reductase	1.072	−1.092
c20978_g4	K00963	13714056	13720625	*FLC/MAF*-like	1.341	−0.997
	K00964	1373553	13741456			
c10648_g1	K00865	10430345	10435526	None	0.809	−1.486
c17440_g1	K00442	2146053	2147623	None	0.637	−1.232
c9036_g1	K00914	11079962	11080712	Heat shock protein 17	1.128	−1.084
c19200_g1	K00366	1733256	1738776	Nucleotide kinase	0.609	−0.746
c20479_g10	K00174	767638	770685	Mg-protoporphyrin IX monomethyl ester (oxidative) cyclase	0.453	−0.568
c20445_g1	K00898	10855024	10858163	Mitochondrial carrier	0.268	−0.559
c20726_g3	K00680	5122223	5124278	Oligopeptidase	0.434	−0.526
c17229_g1	K00920	11148447	11149300	Glutamate–ammonia ligase	−0.292	0.601
c5465_g1	K00628	3367069	3367984	20S proteasome subunit beta 2	−0.305	0.749
c19630_g1	K01332	23352181	23352990	Voltage-dependent anion channel protein 1	−0.318	0.527
c18381_g1	K01482	24129171	24130294	Mitochondrial phosphate carrier protein	−0.375	0.498
c16447_g1	K01277	22948375	22949824	WD40 REPEAT PROTEIN 12, 37	−0.438	0.530
c17701_g1	K00101	470308	472339	Nuclear pore complex protein Nup93	−0.559	0.631
c6503_g1	K01402	23807463	23807463	Pentatricopeptide repeat	−0.810	1.782
c6392_g1	K00707	5352960	5353641	Urease	−0.970	1.462

QTL, quantitative trait loci; LG, linkage group.

### SVP, but not FLC/MAF, gene expression correlates with vernalization responsiveness

To confirm that expression of the four major flowering time candidates matched predictions for population differences in vernalization responsiveness, qPCR analyses were carried out on several *SVP* and *FLC/MAF* genes located in the previously defined vernalization QTL (Friedman and Willis 2013). For *SVP*-like genes, Phytozome BLAST searches, and maximum likelihood phylogenetic analyses, identified six distinct paralogs (Table S1 and Figure S3A), three of which (H02293, H02296, and H02298) are located in the QTL on LG 8. Gene-specific *SVP* primers designed from the IM62 sequences reliably amplified distinct amplicons from either shoot apex cDNA (*SVP* H02293 and *SVP* H02296), or genomic DNA (H02298) of LMC24 plants (Figure S3A). Similar analyses for *FLC/MAF*-like genes identified 13 paralogs, 12 of which are located in the vernalization QTL on LG 11 (Table S1 and Figure S3B). Phylogenetic analyses revealed two major clades of *M. guttatus FLC/MAF*-like genes, each having high levels of sequence similarity suggestive of recent duplication events. Orthology of all newly sequenced LMC24 candidate genes was clear, and well supported relative to distinct IM62 loci (Figure S3B). However, we failed to amplify orthologs of *FLC/MAF* K00961 (clade I), *FLC/MAF* K00963 (clade II), *FLC/MAF* K00965 (clade II), *FLC/MAF* K00984 (clade II), *FLC/MAF* K00994 (clade II), and *FLC/MAF* K00996 (clade II), due to low expression, lack of sampling, or gene loss; *FLC/MAF K01001* was not targeted because it is truncated in the reference genome.

**Figure 4:**
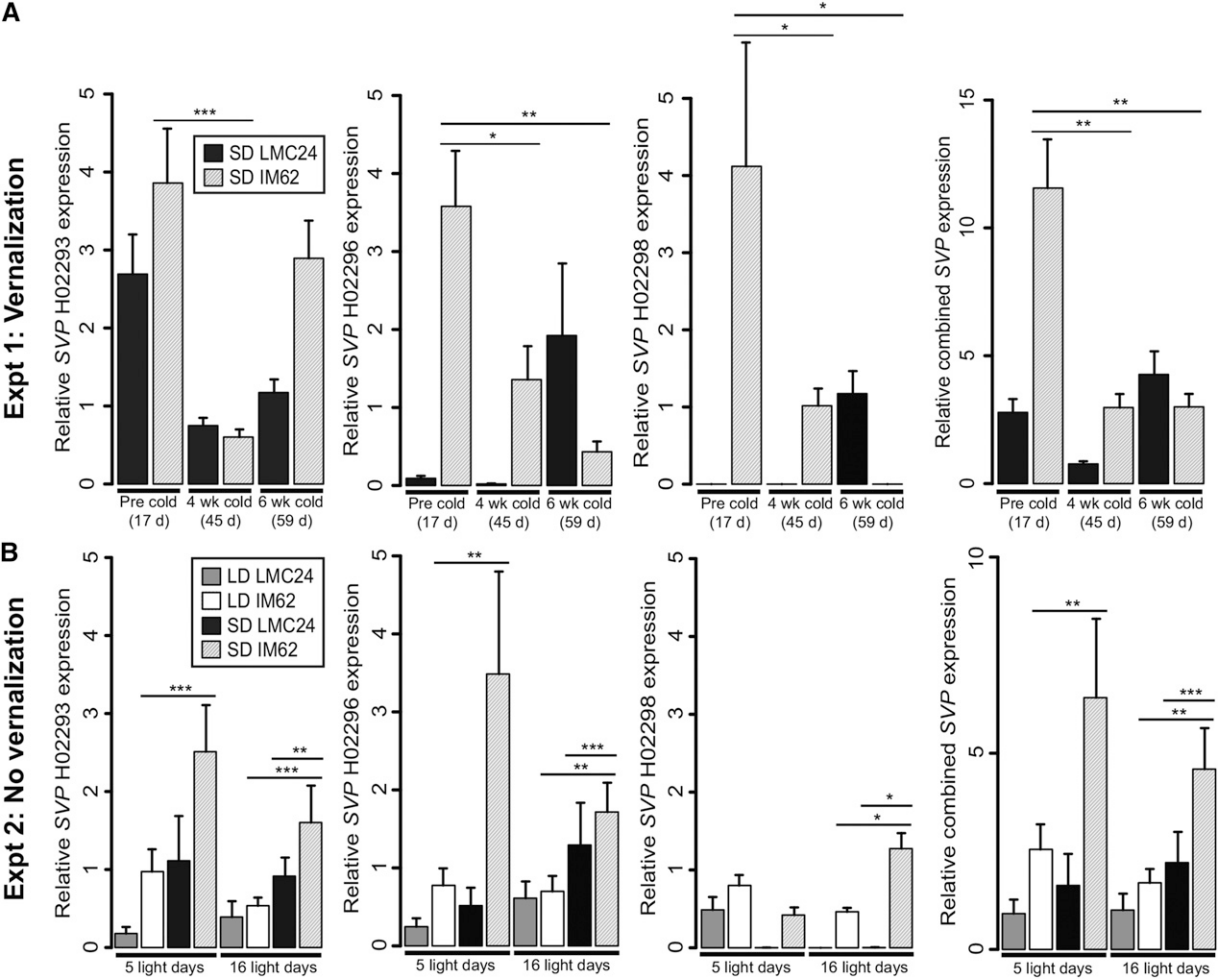
Transcriptional responses of *SVP*-like putative flowering time repressors. (A) Relative *SVP*-like gene expression in response to temperature and population in experiment 1. Samples are matched by chronological time of growth. (B) Relative *SVP*-like gene expression in response to temperature, population, and number of light days in experiment 2. Bars show means for four to five biological replicates with associated standard deviations. Horizontal lines span pairwise comparisons, with asterisks indicating significant pairwise differences using a Tukey test (*** *P* < 0.001; ** *P* < 0.01; * *P* < 0.05).

The candidate *SVP*-like genes include two that were differentially expressed and located within the QTL, and an additional one located within the QTL that was undetected in the transcriptome analysis. qPCR analyses demonstrated that log-transformed expression of each gene, individually and combined, significantly decreased with vernalization for IM62 in experiment 1 (Tukey test *P* < 0.05) (Figure 4A), confirming the RNAseq results. In experiment 2, where sampling was controlled for light hours between photoperiod treatments, all predictions for daylength responsive repressors were met for *SVP* H02293 and H02296 individually, and all three genes together (Figure 4B). In particular, transcripts of *SVP* H02293 and H02296 were significantly higher in IM62 plants grown with an initial period of repressive short-days compared with plants grown only under inductive long-days (*P* < 0.001 and *P* < 0.01, respectively). The expression of *SVP* H02293 and H02296 was also significantly higher in IM62 *vs.* LMC24 plants that received an initial period of short-days and were transferred back to long-days (*P* < 0.01 and *P* < 0.001, respectively). This latter observation is consistent with the hypothesis that flowering time is stably repressed by *SVP*-like genes from short-days to long-days in IM62, but not LMC24. For *SVP* H02298, predictions were met for photoperiod and population effects after 16 light d (*P* < 0.05), but not for photoperiod effects on IM62 after 5 light d (Figure 4B).

**Figure 5:**
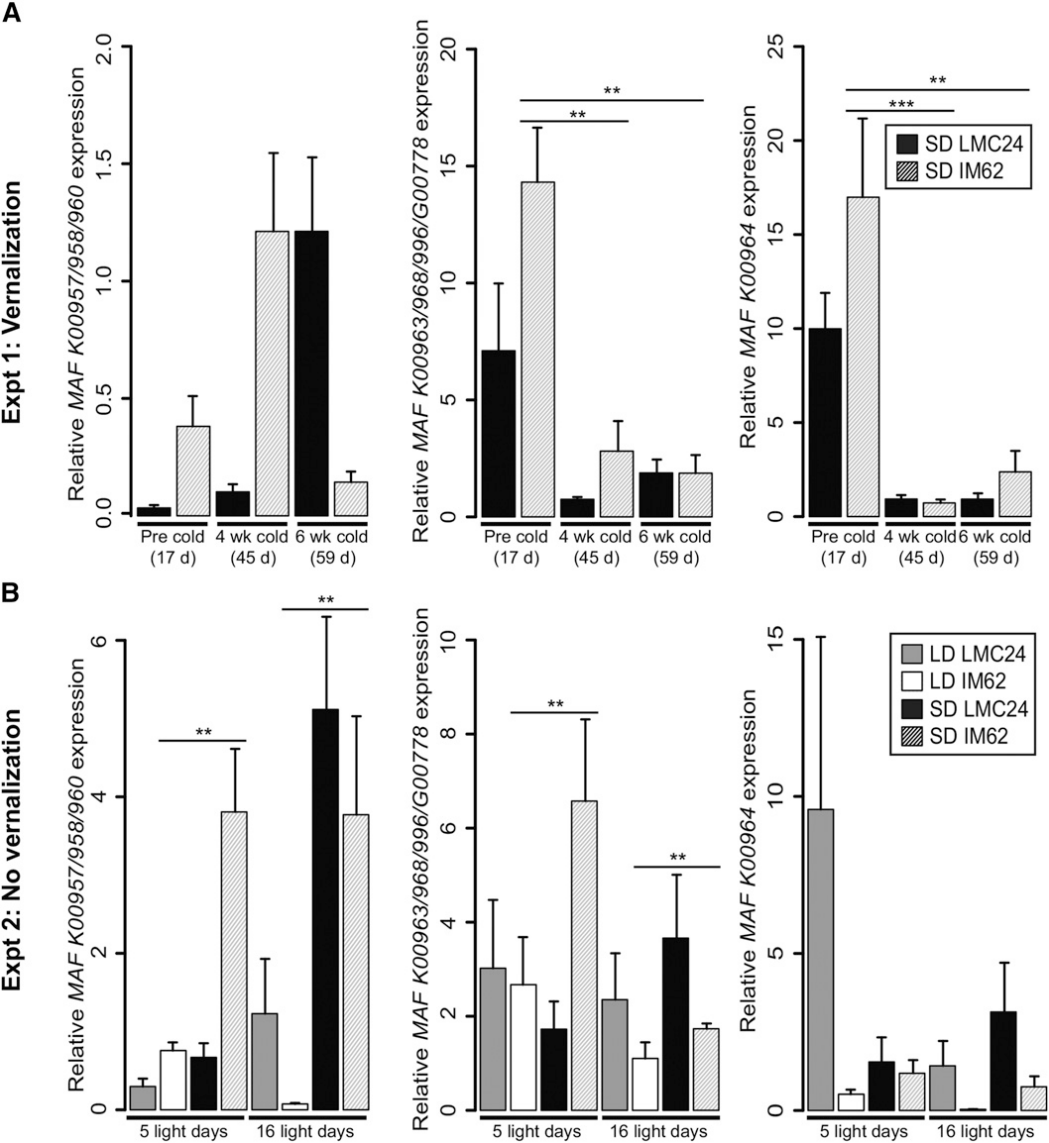
Transcriptional responses of *FLC/MAF*-like putative flowering time repressors. (A) Relative *FLC/MAF*-like gene expression in response to temperature and population in experiment 1. Samples are matched by chronological time of growth. (B) Relative *FLC/MAF*-like gene expression in response to temperature, population, and number of light days in experiment 2. Bars show means for four to five biological replicates with associated standard deviations. Horizontal lines span pairwise comparisons, with asterisks indicating significant pairwise differences using a Tukey test (*** *P* < 0.001; ** *P* < 0.01).

Two *FLC/MAF*-like genes (clade II *FLC/MAF* K00963 and *FLC/MAF* K00964) showed differential expression patterns in the IM62 RNAseq experiment consistent with predictions for vernalization responsive flowering repressors. Nonetheless, we assessed the expression of multiple, closely related *FLC/MAF* genes using qPCR. Consistent with transcriptome analyses, primers simultaneously targeting three closely related clade I *FLC/MAF* genes (K00957, K00958, and K00960) revealed that these genes are not consistently downregulated with vernalization in IM62 (experiment 1) (Figure 5A), and that there is no population difference in expression after transfer from nonpermissive to permissive photoperiod conditions in experiment 2 (Figure 5B). Amplification of candidate genes K00963 and K00964 (the former in combination with closely related paralogs K00968, K00996, and G00778) verified that both genes are downregulated with vernalization in IM62 (*P* < 0.01) as shown in the RNAseq experiment (Figure 5A). However, counter to predictions, K00963 and K00964 expression was not higher in IM62 *vs.* LMC24 plants grown under short-days and transferred back to long-days (Figure 5B).

## Discussion

Previous studies in *M. guttatus* identified two major QTL underlying population differences in short-day mediated vernalization responsiveness (Friedman and Willis 2013). These QTL together harbor 20 putative flowering time genes, and several hundred genes from other GO categories. To determine major genetic pathways that might underlie vernalization responsiveness in IM62, we identified genes that were differentially expressed between photoperiod treatments and following vernalization, including those involved in metabolism, hormone signaling, stress responsiveness, and flowering time. Of the 346 candidate genes, 36 fell into the vernalization QTL regions, four of which were annotated as flowering time genes belonging to the *SVP* and *FLC/MAF* MADS-box transcription factor subfamilies. Follow-up gene expression analyses validated the response of IM62 *SVP*- and *FLC/MAF*-like candidate genes to photoperiod and cold, but only the *SVP*-like genes showed population variation in expression that correlated with flowering time. Together with functional studies of *SVP*-like genes in other species, these data are consistent with *SVP*-like genes being repressors of flowering that have been coopted for short-day mediated vernalization in IM62.

*Arabidopsis* SVP has been implicated in both temperature- and photoperiod-dependent flowering pathways, where it forms a complex with related temperature-responsive proteins FLC and FLM, resulting in repression of *FT* and flowering (Searle *et al.* 2006; Lee *et al.* 2013; Posé *et al.* 2013). Under long-day photoperiods, SVP also reduces expression of the flowering promoter miR172 (Jung *et al.* 2007). Thus, the developmental function of SVP is determined by both temperature and photoperiod. In the *M. guttatus* vernalization responsive IM62 population, total transcription of the three linkage group 8 *SVP*-like genes was reduced in response to vernalization. These data are consistent with conservation of negative flowering regulation among *M. guttatus* linkage group 8 *SVP* genes, *A. thaliana SVP*, and *Antirrhinum majus INCOMPOSITA* (*INCO*) (Masiero *et al.* 2004; Li *et al.* 2008; Gregis *et al.* 2013).

The observed expression pattern of *SVP*-like genes in IM62 leads us to hypothesize that decreased expression of *SVP*-like genes during vernalization negates the repressive effects of short-day photoperiods. In addition, the expression of candidate *SVP*-like genes in plants grown without vernalization meets our predictions for a gene directly or indirectly involved in flowering differences between the Californian (LMC) and Oregon (IM) populations. Assuming they are indeed repressors of flowering, the significant difference in expression between short-day exposed IM plants and all other treatments is consistent with *SVP*-like genes being among the causative loci for population differences in vernalization response. Alternatively, the difference in expression could be explained by evolution of an upstream regulator. Promoter swapping studies between IM and LMC *SVP* alleles expressed in different genetic backgrounds of *M. guttatus* will be required to discriminate between these alternative hypotheses. However, we currently favor the former hypothesis for several reasons.

First, none of the known circadian clock regulators of *SVP*-like genes (Yoshida *et al.* 2009; Nefissi *et al.* 2011) were found in the QTL of interest. Second, it was recently discovered that a single amino acid substitution causing loss of SVP functionality was responsible for early flowering of particular *A. thaliana* accessions under short-day conditions (Méndez-Vigo *et al.* 2013). Finally, the genomic region containing *SVP* H02293, H02296, and H02298 has been identified as a major QTL underlying flowering time differences, and critical photoperiod, in multiple *Mimulus* crosses (Hall *et al.* 2010; Friedman and Willis 2013; Fishman *et al.* 2014; Zuellig *et al.* 2014). This latter finding suggests that *SVP*-like genes might have been the target of repeated diversifying selection in different populations and species of *Mimulus*.

Despite the evidence supporting a role for *SVP* genes, we cannot discount the possibility that genes hitherto lacking a role in flowering are involved in the evolution of IM62 vernalization responsiveness. Although the *SVP* H02293 and H02296 genes are our strongest candidates, 342 additional genes were short-day vernalization responsive, and fell within vernalization QTL from a mapping population between IM62 and LMC24 (Friedman and Willis 2013). Moreover, since our study focused on changes at the level of gene expression, any structural protein coding changes involved in flowering time evolution would only be indirectly detected through their impacts (if any) on downstream transcriptional targets. Thus, to further test the importance of *SVP*-like allelic variation in flowering time evolution, we suggest future studies incorporating fine-scale mapping, comparative transcriptomics, and reverse genetics.

In summary, our data suggest that evolution in the regulation of *SVP*-like genes has been a critical step in the adaptation of *M. guttatus* to seasonally cold environments. Recruitment of flowering repressors in temperature-mediated developmental transitions has previously been described in cereal grasses (Yan *et al.* 2004) and *A. thaliana* (Gu *et al.* 2013). However, in each case, the genes under selection are phylogenetically distinct (Preston and Sandve 2013). The emerging picture then, is that the evolution of vernalization responsiveness is not constrained by the genetic architecture of a common flowering time pathway, but is convergent in terms of both the trait and its underlying genes. These results have implications for past and future adaptation of flowering plants to changing climates, and indicate that adaptation to temperate climates might be less difficult than once thought.

## Supplementary Material

Supplemental Material
