# Supplementary material for: Comparative Transcriptomics Indicates a Role for SHORT VEGETATIVE PHASE (SVP) Genes in Mimulus guttatus Vernalization Response
Source: G3 (Bethesda). 2016 Feb 25;6(5):1239–49. doi: 10.1534/g3.115.026468 (PMC4856076; doi:10.1534/g3.115.026468)
Supplement: Supplemental Material [file supp_6_5_1239__index.html]

Comparative Transcriptomics Indicates a Role for SHORT VEGETATIVE PHASE (SVP) Genes in Mimulus guttatus Vernalization Response — Supplemental Material 

# Comparative Transcriptomics Indicates a Role for *SHORT VEGETATIVE PHASE* (*SVP*) Genes in *Mimulus guttatus* Vernalization Response

## Supplemental Material for Preston *et al.*, 2016

**Files in this Data Supplement:**

- Table S1 - Vernalization QTL candidate flowering time genes identified in Friedman and Willis (2013) and their corresponding contigs from the RNAseq experiment. (.pdf, 15 KB)
- Table S2 - Primers used for qRT-PCR. (.pdf, 72 KB)
- Table S3 - Summary of general linear models of the influences on leaf number, leaf length, and days to flowering. (.pdf, 111 KB)
- Figure S1 - Boxplots of days to flowering for *M. guttatus* IM62 in response to (a) warm short-days and (b) cold short-days. (.eps, 281 KB)
- Figure S2 - Heatmaps showing the 100 most highly differentially expressed genes between RNA extracted from short-day 6 week vernalized (SD 6 wk C), short-day "pre-cold" 17 d warm treated (SD 17 d W), and long-day "pre-cold" 17 d warm (LD 17 d W) treated plants. The most prevalent gene ontology categories are shown for genes that show relatively high or low expression with vernalization (SD 6 wk C) versus pre-cold. R1 and R2 denote biological replicates 1 and 2, respectively. (a) IM62 genes. (b) LMC24 genes. (.eps, 1,002 KB)
- Figure S3 - Maximum likelihood phylogenetic analyses of candidate *SVP*- and *FLC/MAF*-like genes and homologs from core eudicots. (a) *Mimulus guttatus* IM62 has six orthologs of *Arabidopsis thaliana SVP* derived from recent duplication events, three of which are located in the vernalization QTL region on linkage group 8. *M. guttatus* LMC24 has a clear ortholog for each candidate gene. (b) *Mimulus guttatus* IM62 has 13 orthologs of *Arabidopsis FLC/MAF* genes, 12 of which are located in the vernalization QTL region on linkage group 11, and one of which is predicted to be a truncated pseudogene (grey box). Extensive amplicon sequencing from genomic and cDNA identified four *FLC/MAF*-like genes in LMC24. The other copies are inferred to have been lost from the genome, degenerated, or expressed below the level of standard PCR detection. Genes that were significantly downregulated in response to vernalization following initial warm short-day, but not long-day, conditions are highlighted in bold. Maximum likelihood bootstrap values above 70% are associated with each branch where applicable. Asterisks denote bootstrap values of 100%. (.eps, 361 KB)
