# Supplementary material for: Comparative Transcriptomics Indicates a Role for SHORT VEGETATIVE PHASE (SVP) Genes in Mimulus guttatus Vernalization Response
Source: G3 (Bethesda). 2016 Feb 25;6(5):1239–49. doi: 10.1534/g3.115.026468 (PMC4856076; doi:10.1534/g3.115.026468)
Supplement: Supplemental Material [file supp_g3.115.026468_TableS1.pdf]

Table S1. Vernalization QTL candidate flowering time genes identified in Friedman and Willis (2013) and their corresponding contigs from the RNAseq experiment.

Gene	Phytozome ID	Transcriptome
CONSTANS-like 2	mgv1a1009529	c16304_g1
CONSTITUTIVE PHOTOMORPHOGENIC 1	H01246	Not found
FLOWERING LOCUS C	K00957	Not found
	K00958	Not found
	K01001	Not found
GA REQUIRING 1	mgv1a 020029	Not found
GIBBERELLIC ACID INSENSITIVE	mgv1a 020653	Not found
	H02266	Not found
	K00894	Not found
GIBBERELLIN 2 OXIDASE 2	mgv1a021466	Not found
	H02079	Not found
	H01362	Not found
GIBBERELLIN 2 OXIDASE 8	H01552	Not found
GIBBERELLIN 20 OXIDASE 2	H00683	c31600_g1
		c33341_g1
		c33724_g1
MADS AFFECTING FLOWERING 1	K00968	Not found
	K00996	Not found
MADS AFFECTING FLOWERING 3	K00963	Not found
MADS AFFECTING FLOWERING	K00964	C20978_g4
PHYTOCHROME DEFECTIVE C	H01285	Not found
PHYTOCHROME DEFECTIVE E	H01380	Not found
PHYTOCHROME KINASE SUBSTRATE 4	K00204	c5003_g1
		c20681_g1
		c20681_g7
		c31523_g1
RECEPTOR-LIKE KINASE IN FLOWERS 1	H01401	Not found
REDUCED VERNALIZATION RESPONSE 1	H02193	c12302_g1
SERRATED LEAVES AND EARLY FLOWERING	H00695	Not found
SHORT VEGETATIVE PHASE	H02293	c20784_g10
	H02296	Not found
	H02298	c20784_g10
SPA1-RELATED 4	H01941	c20260_g2
SQUAMOSA PROMOTER BINDING PROTEIN-like 9	K00051	c17930_g1

		c18890_g5
SUPPRESSOR OF PHYB-4#3	H02315	Not found
	K00494	
ZEITLUPE	K00230	Not found
