# Supplementary material for: Comparative Transcriptomics Indicates a Role for SHORT VEGETATIVE PHASE (SVP) Genes in Mimulus guttatus Vernalization Response
Source: G3 (Bethesda). 2016 Feb 25;6(5):1239–49. doi: 10.1534/g3.115.026468 (PMC4856076; doi:10.1534/g3.115.026468)
Supplement: Supplemental Material [file supp_g3.115.026468_TableS2.pdf]

Table S2. Primers used for qRT-PCR.

GENE	FORWARD PRIMER (5' to 3')	REVERSE PRIMER (5' to 3')	EFFICIENCY (%)
SVP.254	AGCGAATGTGTGCAACTCAG	TGCATTCATCCACTGTAAGGC	96
SVP.256	GCGGCAGTAAAACGGGAAC	GAGGACCAGCTGAGTTGC	92
SVP.258	GGTGGCGGTAGTAAAACGAG	GAGGACCAGCTGAGTTGC	93
MAF.K00960	AGGGGCAGTCAAACAACTCT	CTTCTGCCAGGCTCTTTTCC	93
MAF.K00958	Same as *MAF.K00960*	Same as *MAF.K00960*	
MAF.K00957	Same as *MAF.K00960*	Same as *MAF.K00960*	
MAF.K00968	GTTGACCAGCTAAGTGTGGC	TCTTGGTCGAACGTATCTCCA	93
MAF.G00778	Same as *MAF.K00968*	Same as *MAF.K00968*	
MAF.K00996	Same as *MAF.K00968*	Same as *MAF.K00968*	
MAF.K00963	Same as *MAF.K00968*	Same as *MAF.K00968*	
MAF.K00964	AGCATACAACACCGGACCTC	TCAAGCAGCTTACACCATGC	102
