# Supplementary material for: Comparative Transcriptomics Indicates a Role for SHORT VEGETATIVE PHASE (SVP) Genes in Mimulus guttatus Vernalization Response
Source: G3 (Bethesda). 2016 Feb 25;6(5):1239–49. doi: 10.1534/g3.115.026468 (PMC4856076; doi:10.1534/g3.115.026468)
Supplement: Supplemental Material [file supp_g3.115.026468_TableS3.pdf]

Table S3. Summary of general linear models of the influences on leaf number, leaf length, and days to flowering.

Source of variation	Leaf number	First true leaf length	Days to flowering
**Experiment 1: Vernalization**			
Photoperiod	$F_{1,108} = 174.26$ $P < 0.001$	$F_{1,127} = 372.05$ $P < 0.001$	$F_{1,315} = 31.93$ $P < 0.001$
Population	$F_{1,108} = 18.16$ $P < 0.001$	$F_{1,127} = 480.35$ $P < 0.001$	$F_{1,315} = 243.78$ $P < 0.001$
Time	$F_{1,108} = 6.88$ $P = 0.002$	$F_{3,127} = 64.93$ $P < 0.001$	Not applicable
Photoperiod x Population	$F_{1,108} = 11.77$ $P < 0.001$	$F_{1,127} = 0.16$ $P = 0.694$	$F_{1,315} = 0.43$ $P = 0.514$
Photoperiod x Time	$F_{1,108} = 7.27$ $P = 0.001$	$F_{3,127} = 5.97$ $P < 0.001$	Not applicable
Population x Time	$F_{1,108} = 1.83$ $P = 0.166$	$F_{3,127} = 0.35$ $P = 0.792$	Not applicable
Photoperiod x Population x Time	$F_{1,108} = 5.93$ $P = 0.004$	Not applicable	Not applicable
**Experiment 2: No vernalization**			
Photoperiod	$F_{1,72} = 152.03$ $P < 0.001$	$F_{1,90} = 202.32$ $P < 0.001$	$F_{1,305} = 7905$ $P < 0.001$
Population	$F_{1,72} = 6.08$ $P = 0.016$	$F_{1,90} = 115.68$ $P < 0.001$	$F_{1,305} = 3083$ $P < 0.001$
Time	$F_{1,72} = 452.68$ $P < 0.001$	$F_{3,90} = 394.6$ $P < 0.001$	Not applicable
Photoperiod x Population	$F_{1,72} = 54.73$ $P < 0.001$	$F_{1,90} = 11.10$ $P = 0.001$	$F_{1,305} = 63086$ $P < 0.001$
Population x Time	$F_{1,72} = 53.76$ $P < 0.001$	$F_{3,90} = 16.27$ $P < 0.001$	Not applicable
